# Surfactant-Assisted Label-Free Fluorescent Aptamer Biosensors and Binding Assays

**DOI:** 10.3390/bios13040434

**Published:** 2023-03-29

**Authors:** Hanxiao Zhang, Albert Zehan Li, Juewen Liu

**Affiliations:** Department of Chemistry, Waterloo Institute for Nanotechnology, University of Waterloo, Waterloo, ON N2L 3G1, Canada

**Keywords:** aptamers, biosensors, surfactant, fluorescence

## Abstract

Using DNA staining dyes such as SYBR Green I (SGI) and thioflavin T (ThT) to perform label-free detection of aptamer binding has been performed for a long time for both binding assays and biosensor development. Since these dyes are cationic, they can also adsorb to the wall of reaction vessels leading to unstable signals and even false interpretations of the results. In this work, the stability of the signal was first evaluated using ThT and the classic adenosine aptamer. In a polystyrene microplate, a drop in fluorescence was observed even when non-binding targets or water were added, whereas a more stable signal was achieved in a quartz cuvette. Equilibrating the system can also improve signal stability. In addition, a few polymers and surfactants were also screened, and 0.01% Triton X-100 was found to have the best protection effect against fluorescence signal decrease due to dye adsorption. Three aptamers for Hg^2+^, adenosine, and cortisol were tested for their sensitivity and signal stability in the absence and presence of Triton X-100. In each case, the sensitivity was similar, whereas the signal stability was better for the surfactant. This study indicates that careful control experiments need to be designed to ensure reliable results and that the reliability can be improved by using Triton X-100 and a long equilibration time.

## 1. Introduction

Developing assays to study aptamer binding is of great importance to validate newly selected aptamers, characterize aptamer binding, and design biosensors [1,2,3,4,5]. Among the numerous aptamer binding assays [6,7,8,9], those based on DNA staining dyes such as SYBR Green I, thioflavin T, and thiazole orange have been very popular [10,11,12,13,14,15] due to their label-free nature and cost-effectiveness. In a typical assay, a dye and an aptamer are mixed, and their fluorescence is monitored as a function of the target analyte concentration. The assumption is that aptamer–target binding can either promote dye binding or displace the dye, leading to increased or decreased fluorescence, respectively [13]. Another popular label-free method is to use citrate-capped gold nanoparticles. However, our recent studies indicated that gold nanoparticles can strongly adsorb various target molecules, which can mislead the interpretation of assay results [16,17]. In this regard, the dye-staining assay is relatively more reliable.

However, the dye-staining assay also has complications. First, it may not work for all aptamers and the applicability of the assay is a trial-and-error process. Sometimes, the change in fluorescence is very small, which can compromise the reliability of this method and make quantitative measurement difficult. For many such assays, the fluorescence intensity drops when target molecules are added and the change in fluorescence intensity is within one-fold, such as in the adenosine aptamer with ThT [18,19,20,21]. Therefore, it is important that the observed fluorescence drop is due to aptamer binding. To ensure this, it is critical to ensure a stable fluorescence signal before titrating target molecules. Since DNA staining dyes are mostly cationic, they can be adsorbed by sample containers such as glass cuvettes and plastic microplates that have negatively charged surfaces [22,23]. This type of adsorption interaction may strongly interfere with the assay and even cause false signals.

In this study, we studied the signal stability problem and explored two methods to establish stable background fluorescence. The first method is simply to wait for the signal to stabilize before titrating target molecules, where we observed time-dependent signal change even without adding target molecules. The second method is to add surfactants to cover the surface of reaction vessels. For this, we screened various surfactants and demonstrated the stabilization effect of Triton X-100 for a few representative aptamers.

## 2. Materials and Methods

### 2.1. Chemicals

All of the DNA samples were ordered from Integrated DNA Technologies (Coralville, IA). The DNA sequences and modifications used are in Table 1. Surfactant, mercury acetate, SYBR Green I (SGI), thioflavin T (ThT), adenosine (ADE), and cortisol were purchased from Sigma-Aldrich. Half-area black 96-well microplates and human AB serum were purchased from Corning and Greiner. Quartz fluorescence cuvettes were purchased from Agilent. Several buffers were prepared. Buffer 1 (for most adsorption studies and Ade aptamer): 50 mM pH 7.6 Tris, 500 mM NaCl, 20 mM MgCl_2_. Buffer 2 (for T_30_ Hg^2+^ binding DNA): 20 mM pH 7.5 MOPS. Buffer 3 (for CSS.1-42 aptamer): 20 mM pH 7.5 HEPES, 100 mM NaCl, 10 mM MgCl_2_. Buffer 4 (for Ade and Ade-M2 aptamer): 10 mM HEPES buffer, pH 7.6, 50 mM NaCl, and 4 mM MgCl_2_. Buffer 5 (for Ade aptamer): 10 mM HEPES buffer, pH 7.6, 50 mM NaCl, 4 mM MgCl_2_, and 5% human AB serum.

### 2.2. Effect of Time-Dependent Stabilization

Time-dependent stabilization was evaluated using a 96-well black plate. The sample solutions contained 100 nM adenosine aptamer or Ade-M2 aptamer and 2 μM ThT in Buffer 4 or Buffer 5 (when testing the effect of 5% serum on the titration of the adenosine aptamer by adenosine). Fluorescence intensity was monitored at 420 nm excitation and 490 nm emission using a Horiba Fluoromax 4 fluorometer (500 μL, cuvette-based) maintained at 20°C by a water bath or a Tecan Spark plate reader (100 μL, plate-based). Adenosine, guanosine, and MilliQ water were gradually titrated up to a final target concentration of 500 μM. Titration was either performed immediately or equilibrated by waiting for 10 min to reach a stable baseline before titration.

### 2.3. Test of Different Surfactants and Assay Materials

The protective effects of different surfactants were evaluated using a 96-well black plate (Corning). The sample solution (100 µL) contained 200 nM DNA1 aptamer, 0.02× SGI in Buffer 1 with different concentrations of Triton X-100 (0.1%, 0.01%, 0.001%, and 0.0001%). The fluorescence intensity was monitored under excitation at 488 nm and absorption at 532 nm for 30 min using a Tecan Spark plate reader. The effects of different assay materials were tested using 96-well black plates (Corning and Greiner) and a quartz cuvette. To test the effect of different surfactants, a 96-well black plate (Corning) and a quartz cuvette were used. For the plate method, 100 µL of the sample solution contained 200 nM DNA1 aptamer, 0.02×SGI in Buffer 1, and 0.01% surfactant (Triton X-100, Tween 20, PEG 2000, PEG 20000, SDS or CTAB). For the cuvette method, 500 µL sample solution contained 1 µM DNA1 aptamer, 0.1× SGI in Buffer 1, and 0.01% of the various surfactants. To test the influence of Triton X-100 on the adsorption of dyes on sample containers, 0.5×SGI in Buffer 1 was incubated for 15 min in a 96-well black plate, and then the solution was fully removed. Next (0.01% Triton X-100 or no Triton X-100) in Buffer 1 was added along with a 70-mer double-stranded DNA into the same wells used initially. In clean wells, 70-mer double-stranded DNA, in the presence of 0.01% Triton X-100, was titrated up to a final concentration of 0.4 µM SGI. Fluorescence intensity was monitored using a Tecan Spark plate reader at 488 nm excitation and 532 nm emission.

### 2.4. Hg^2+^ Sensing Assay

The effect of surfactants on the binding of Hg^2+^ by T_30_ DNA was evaluated using a 96-well black plate (Corning) in Buffer 2 containing 200 nM T_30_ aptamer, 0.1× SGI, and 0% or 0.01% Triton X-100. The sample solution (100 µL) was titrated with mercury acetate up to a final concentration of 3 µM. Fluorescence intensity was monitored using a Tecan Spark plate reader at 488 nm excitation and 532 nm emission. For signal stability measurement, 100 µL of sample solution contained 200 nM T_30_ aptamer, 0.1× SGI, 2 µM mercury acetate, and 0% or 0.01% Triton X-100 in Buffer 2. Fluorescence intensity was monitored using the microplate reader with 488 nm excitation and 532 nm emission for 30 min.

### 2.5. Adenosine Sensing Assay

The effect of surfactants on the binding of adenosine by its aptamer was evaluated using a 96-well black (Corning) plate in Buffer 1 containing 1 µM aptamer, 1 µM ThT, and 0% or 0.01% Triton X-100. The sample solution (100 µL) was titrated with adenosine up to a final concentration of 150 µM. Fluorescence intensity was monitored using the microplate reader at 488 nm excitation and 532 nm emission. For signal stability measurement, 100 µL of sample solution contained, 1 µM aptamer, 100 µM adenosine, 1 µM ThT, and 0% or 0.01% Triton X-100 in Buffer 1. Fluorescence intensity was monitored using the microplate reader with 488 nm excitation and 532 nm emission for 30 min.

### 2.6. Cortisol Sensing Assay

The effect of surfactants on the binding of cortisol to CSS.1-42 DNA was evaluated using a 96-well black plate (Corning) in Buffer 3 containing 1 µM CSS.1-42 aptamer, 0.1× SGI, and 0% or 0.01% Triton X-100. The sample solution (100 µL) was titrated with cortisol up to a final concentration of 6 µM. Fluorescence intensity was monitored using the microplate reader at 488 nm excitation and 532 nm emission. For signal stability measurement, 100 µL sample solution contained 1 µM CSS.1-42 aptamer, 5 µM cortisol, 0.1× SGI, and 0% or 0.01% Triton X-100 in Buffer 3. Fluorescence intensity was monitored using the microplate reader at 488 nm excitation and 532 nm emission for 30 min.

## 3. Results and Discussion

### 3.1. Effect of Stabilization in Microplates and Cuvettes

**Figure 1:**
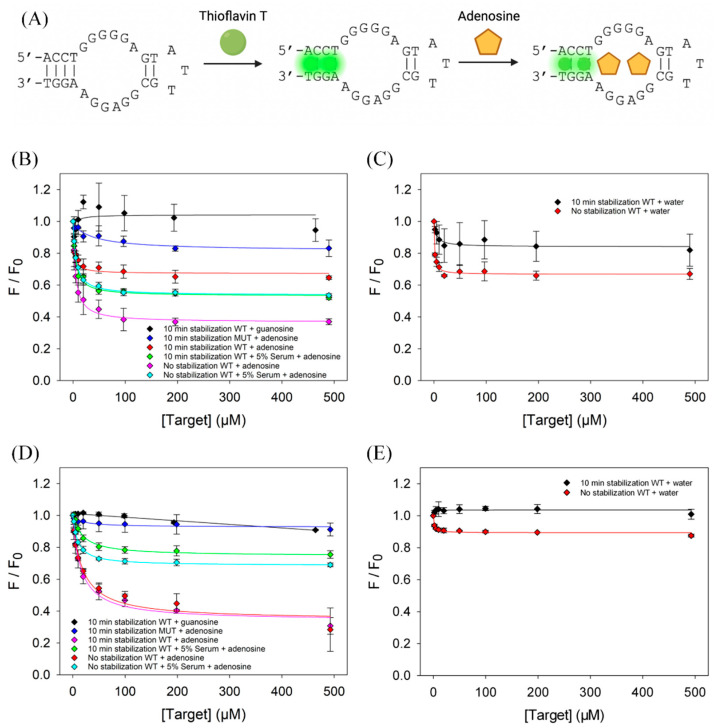
(**A**) Scheme of adenosine binding to the adenosine aptamer detected by ThT. Initially, ThT binds to the aptamer, producing a strong fluorescence signal. Adenosine binding displaces ThT, resulting in a decrease in fluorescence. Binding assay in a polystyrene microplate by (**B**) titrating adenosine and guanosine, and (**C**) titrating water. Binding assay in a quartz cuvette by (**D**) titrating adenosine and guanosine, and (**E**) titrating water. WT: wild-type adenosine aptamer; MUT: Ade Apt M2 mutant. Fluorescence intensities were normalized to their respective baseline value (absent of target) before titration.

The classic adenosine aptamer stained by ThT was used to study the effect of time-dependent signal stability on the dye staining method [24,25]. A schematic representation of the reaction is shown in Figure 1A. In this case, adenosine binding can displace ThT, resulting in decreased fluorescence [18]. In contrast, non-binding molecules do not displace ThT and no fluorescence drops are expected. For this assumption to be valid, it is important that the fluorescence change is only due to aptamer binding instead of other processes, such as dye adsorption by the assay vessel.

First, we conducted the titration in a 96-well microplate without waiting (adenosine was titrated immediately after adding the aptamer and ThT mixture). Adding adenosine produced a 65% decrease in this case (magenta line, Figure 1B). Interestingly, only a 35% drop was obtained after incubating the aptamer and ThT for 10 min in the microplate before titration (red line, Figure 1B). However, when the same titration was conducted in 5% serum, a 45% drop was exhibited with and without incubation (green and turquoise lines, Figure 1B). The result suggested that the serum proteins might have blocked the container surface to prevent further adsorption of the dye.

To ensure that the observed fluorescence drop was specific, we performed a control experiment using a mutant (see Table 1 for the sequence named Ade Apt M2, where two guanines of Ade Apt were mutated to thymines) [18], in which only a 15% drop was observed after stabilization (blue line, Figure 1B). In another control experiment, guanosine was added to the wild-type aptamer, and no change in fluorescence was observed (black line, Figure 1B). Guanosine is known to be a nonbinding molecule for this aptamer [24]. This set of control experiments indicated that the aptamer was specifically bound to adenosine.

We then determined the fitted *K*_d_ values. In the microplate, the *K*_d_ of adenosine binding to the aptamer was 5.0 ± 2.1 μM without stabilization and 2.8 ± 3.1 μM with 10 min stabilization. In 5% serum, the *K*_d_ was 5.6 ± 1.1 μM without stabilization and 5.1 ± 2.0 μM with 10 min stabilization. The literature reported *K*_d_ for this aptamer is between 6 and 20 μM adenosine [18,19,20]. Considering the error associated with this method, there was not a significant difference in *K*_d_ with or without stabilization, and because all determined *K*_d_ are only marginally below the lower bound of the reported *K*_d_, this demonstrates that the ThT-based titration method can provide valuable information on the strength of target binding in this case. In addition, the difference in *K*_d_ determined in the presence and absence of 5% serum was negligible, suggesting that this method can retain its accuracy within biological fluids.

As a further control, water was titrated into the aptamer/ThT samples (Figure 1C). The wild-type aptamer titrated with water produced a 20% drop with 10 min stabilization (fitted *K*_d_ = 11 ± 12), compared to a 33% drop without stabilization (fitted *K*_d_ = 1.3 ± 0.3). This is concerning since in theory water does not impact the aptamer binding to ThT and thus no fluorescence drop should have occurred. This suggests that the system had a time-dependent signal change even without adding target molecules and we reason that two processes occurred simultaneously to contribute to the fluorescence drop: dye adsorption and aptamer binding.

With stabilization, the effect of dye adsorption was minimized. However, without stabilization, the interpretation of the results can be misleading. Despite this, it was still possible to differentiate between binding and non-binding targets based on the results in Figure 1B. Quantitative fitting of the data needs to be careful, since even water produced a similar *K*_d_ compared to adenosine.

We then performed the same reactions using a quartz cuvette, which should have lower adsorption because quartz does not have hydrophobic interactions with DNA-staining dyes. In this case, a 60% fluorescence drop was observed upon adenosine binding, regardless of the 10 min stabilization (red and magenta lines, Figure 1D). When the same experiment was performed in 5% serum, there was a 30% drop without stabilization, compared to a 25% drop with stabilization, again suggesting serum proteins may mitigate dye adsorption (green and turquoise lines, Figure 1D). The aptamer titrated with water produced a 13% drop without stabilization and essentially no drop was observed with stabilization (Figure 1E). Using the cuvette method, the *K*_d_ of adenosine binding to the aptamer was 16.6 ± 8.4 μM with stabilization and 17.5 ± 8.7 μM without stabilization. In serum, the *K*_d_ was 20.9 ± 2.7 μM with stabilization and 9.5 ± 2.1 μM without stabilization Using ITC, we obtained a *K*_d_ of 16.4 μM under similar buffer conditions and DNA samples [26]. In addition, all negative controls generated less than a 15% drop in fluorescence and no clear sign of binding (blue and black lines, Figure 1D). Therefore, quartz cuvettes can generate reliable results for quantitative measurement.

### 3.2. Time-Dependent Signal Stability

**Figure 2:**
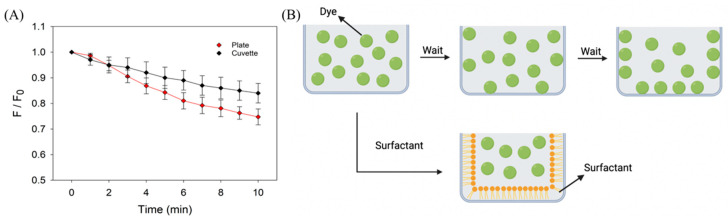
(**A**) Time-dependent fluorescence intensity of the adenosine aptamer and ThT mixture in a polystyrene microplate and a quartz cuvette. (**B**) A scheme showing the adsorption of dyes such as SGI and ThT to the wall of a reaction vessel.

The above studies indicated time-dependent initial fluorescence. We then monitored the kinetics of this initial fluorescence change before titration (Figure 2A). Within 10 min after mixing the aptamer and ThT, the fluorescence in the microplate decreased by 25%, while that in the cuvette decreased by approximately 15%. We attributed this decrease to the adsorption of the dye to the walls of the reaction vessels (Figure 2B). The fluorescence in the cuvette was close to stable at the end of 10 min, but the fluorescence in the microplate continued to decrease. If titration is performed immediately, the observed fluorescence drop is the sum of two processes: aptamer binding and dye adsorption. We also noticed that the amount of fluorescence drop was smaller in the cuvette when guanosine or water was added, compared to the drop seen in Figure 2A, where nothing was added. This could be due to perturbation during addition and mixing, facilitating the systems to equilibrate.

In both the plate- and cuvette-based assays, even with stabilization, there was still a fluorescence drop observed between target concentrations of 0–20 μM for non-binding controls (conditions with guanosine, water, or mutant aptamer). It is extremely important to have appropriate controls so that such non-binding events cannot be interpreted as aptamer binding. Overall, for the adenosine aptamer, it was possible to discern binding targets from non-binding targets using this label-free method. With stabilization and in a non-adsorbing vessel, the measured *K*_d_ values were more reliable. For the adenosine aptamer with ThT, a fluorescence drop of nearly 60% was achieved. However, in other cases, the fluorescence change produced can be much smaller than that achieved using this adenosine aptamer. Careful controls are even more important for those aptamers with smaller signal changes. 

### 3.3. The Protective Effect of Surfactants

Aside from waiting for stabilization, another method to reduce nonspecific adsorption is to make the vessel surfaces less adsorbing. A surfactant is composed of hydrophilic and hydrophobic groups. After adding a surfactant to the solution, the surfactant may adsorb onto the surface, preventing DNA/dye adsorption and stabilizing the fluorescence of the system. Most aptamers can function in the presence of many surfactants [27]. However, surfactants may also interact with DNA and dyes, thereby influencing signal production. To test the effect of surfactants, we first used DNA1 (a 38-mer DNA) and stained it with SGI dye.

**Figure 3:**
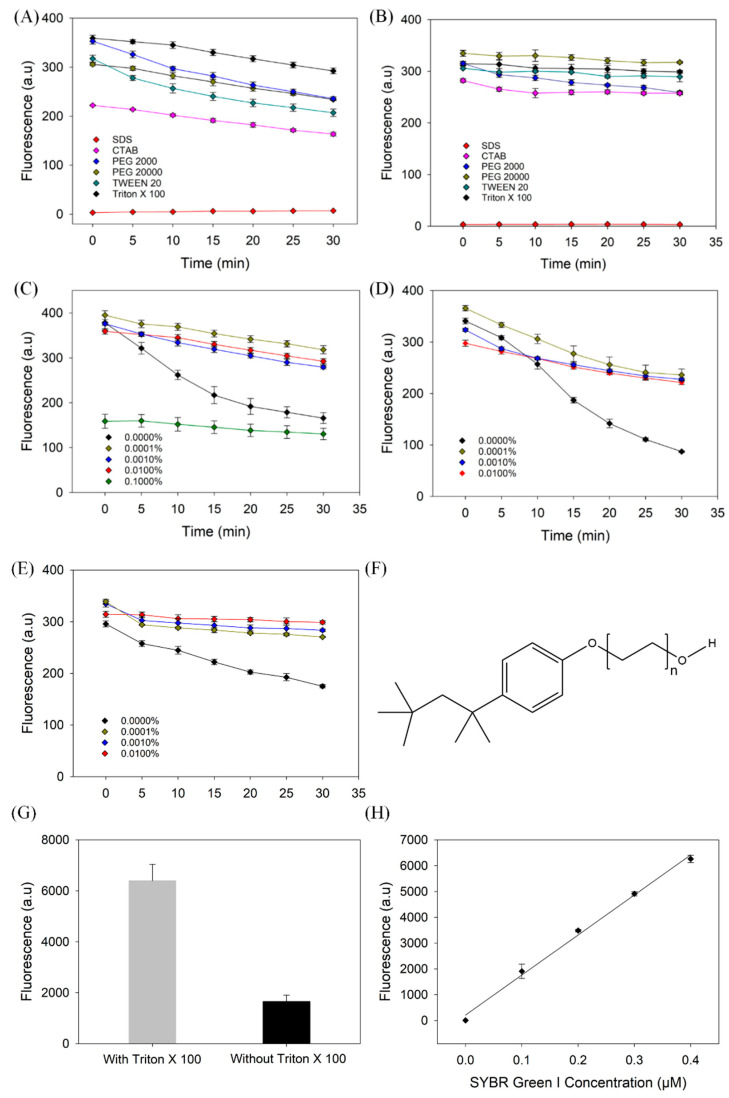
(**A**) Effect of surfactant on signal stability in Corning 96-well microplate. The samples contained 200 nM DNA1, 0.02× SGI, and 0.01% different surfactants in Buffer 1. (**B**) Effect of surfactant on signal stability in a quartz cuvette. The solutions contained 1 µM DNA1, 0.1× SGI, and 0.01% different surfactants in Buffer 1. Effect of Triton X-100 concentration on the stability of fluorescence in (**C**) a Corning microplate, (**D**) a Greiner microplate, and (**E**) a quartz cuvette. (**F**) Structure of Triton X-100. (**G**) Effect of Triton X-100 on SGI pre-stained microplate well containers. Initially, buffer and 1 µM SGI were incubated for 15 min, then solution was removed. To the same wells, buffer with and without 0.01% Triton X-100 was added alongside 70-mer double-stranded DNA. (**H**) Titration curve of 70-mer double-stranded DNA titrated by SGI in the presence of 0.01% Triton X-100.

We first screened a few surfactants and polymers [28]. The basic properties of these surfactants are listed in Table 2. Initially, the fluorescence readings of all surfactant samples were between 200 and 400 fluorescence units (FU) (Figure 3A,B) using both the plate and cuvette, except for SDS (close to 0) and CTAB (lower fluorescence). SDS is an anionic surfactant. We hypothesized that SDS may compete with the aptamer for SGI, resulting in a very weak fluorescence signal. Cationic CTAB may compete with SGI for binding with the DNA. The hydrophilic–lipophilic balance (HLB) values of Triton X-100, CTAB, and SDS are 13.5, 21.4, and 40, respectively [29]. These three surfactants have strong hydrophilicity and weak lipophilic interactions. They mainly bind to DNA and dyes through cation/anion interactions. The data shows that Triton X-100 had the most prominent stabilizing effect on the fluorescence intensity of the DNA-SGI system. Triton X-100 is a non-ionic surfactant (Figure 3F), and thus, the absence of electrostatic interactions with DNA or dye may contribute to its good stabilizing effect.

We then optimized the concentration of Triton X-100. Initially, the fluorescence intensity produced by the different Triton X-100 concentrations was approximately 400 fluorescence units, except for 0.1% Triton X-100, which had an initial fluorescence of 160 units (Figure 3C). We theorize that because the 0.1% concentration exceeds the critical micelle concentration of Triton X-100 (Table 2), the surfactant may sequester a fraction of SGI to decrease the binding of SGI to the DNA, resulting in a decrease in the fluorescence. It was found that 0.01% Triton X-100 produced the lowest rate of fluorescence decrease, while maintaining a stable fluorescence signal.

To further confirm the adsorption of the dyes and the effect of Triton X-100 on dye adsorption by sample containers, we incubated 1 µM SGI with the microplate in buffers. After removing the solutions, we added buffers with or without Triton X-100 at 0.01% and then added a 70-mer double-stranded DNA to measure the fluorescence intensity, which can reflect the level of adsorption of the pre-adsorbed dyes. The samples containing Triton X-100 were 3.9 times more fluorescent than samples without Triton X-100 (Figure 3G), suggesting that Triton X-100 can prevent SGI adsorption. We explain this phenomenon as Triton X-100 can adsorb on the container wall and displace the SGI adsorbed on the container wall. We then titrated samples containing the dsDNA in 96-well plates using SGI (Figure 3H). An amount of 0.4 µM of SGI was needed to achieve 6300 fluorescence. Thus, about 0.6 µM of SGI was adsorbed to the container wall of a well. We calculated that the container walls absorbed approximately 74% of the SGI.

To test this generality, we further monitored the same DNA in a Greiner microplate (Figure 3D) and a quartz cuvette (Figure 3E). Overall, Triton X-100 exhibited a similar stabilization effect. Comparing the different materials, the rate of fluorescence decrease when using the quartz cuvette was lower than that when using the polystyrene plates. For all samples, 0.01% Triton X-100 was found to be the optimal concentration, which was used in subsequent experiments. The molecular weight of Triton X-100 is 646.9 g/mol, and thus its molar concentration was 0.155 mM. This is far greater than the concentration of aptamer (~1 µM). In the following experiments, we used Triton X-100 to further explore the protective effect of surfactants on different aptamers and fluorescent dye conjugates.

### 3.4. Sensing Mercury Using a Thymine-Rich DNA

**Figure 4:**
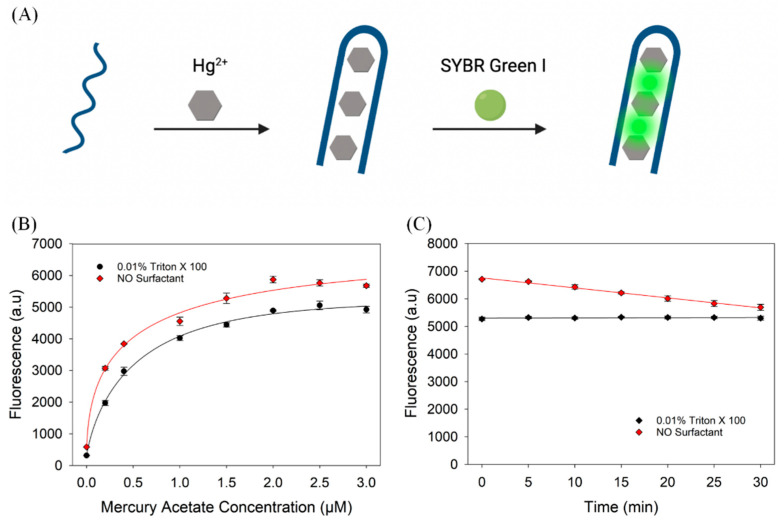
(**A**) A scheme of Hg^2+^ binding to a thymine-rich DNA detected by SGI. Hg^2+^ binds to the DNA and folds into a structure with a long duplex region, resulting in a strong SGI fluorescence. (**B**) Titration curve of 200 nM T_30_ using mercury acetate with and without 0.01% Triton X-100. (**C**) Effect of Triton X-100 on signal stability of T_30_/SGI in the presence of 2 µM Hg^2+^.

We then studied the performance of a few different aptamers under optimized conditions (0.01% Triton X-100). Since the signal variation was more significant in the microplates, we used a microplate for all these experiments. First, we used a T_30_ DNA for the detection of Hg^2+^ ions as shown in Figure 4A [32]. The fluorescence of the system increases significantly upon binding to Hg^2+^. Regardless of the surfactant, the initial and final fluorescence was comparable. Under the protection of Triton X-100, the fluorescence of the system increased 15.6-fold, although the surfactant-free sample also achieved a 10.0-fold increase (Figure 4B). We studied the stability of the fluorescence signal for samples containing 2 µM Hg^2+^ in a 96-well plate for 30 min (Figure 4C). Without Triton X-100, the fluorescence of the system declined almost linearly as the intensity dropped by 15.2% after 30 min. In the presence of 0.01% Triton X-100, the fluorescence of the system did not change. Evidently, Triton X-100 can achieve a more stable signal, which is very important for accurate analytical results.

### 3.5. Evaluation of the Adenosine Aptamer

**Figure 5:**
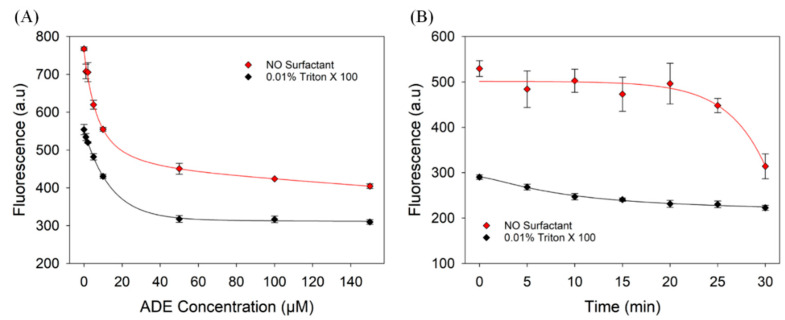
(**A**) Titration curve of 1 µM adenosine aptamer by adenosine without and with 0.01% Triton X-100. (**B**) Effect of Triton X-100 on signal stability of the adenosine aptamer/ThT system in the presence of 100 µM adenosine.

The Hg^2+^ binding DNA has a uniquely strong fluorescence enhancement, which is not seen in most other aptamers. For the majority of aptamers, the change in fluorescence is only within one-fold. Thus, we further tested a few small molecule binding aptamers. We first used adenosine aptamer DNA and ThT to detect adenosine. As shown in Figure 1A, the fluorescence of the system decreased after the addition of adenosine. Triton X-100 had no obvious effect on the fold change in the fluorescence of the system, although the overall fluorescence was approximately 30% lower in the presence of the surfactant (Figure 5A). Next, we monitored the fluorescence kinetics of the samples containing adenosine aptamer and 100 µM adenosine for 30 min (Figure 5B). In the absence of Triton X-100, the fluorescence of the system decreased slowly before 20 min, and then decreased rapidly after 20 min. After 30 min, the fluorescence signal dropped by 41%. The fluorescence of the system did not change significantly after 15 min in the presence of 0.01% Triton X-100. Thus, the addition of Triton X-100 can improve signal reliability in this case. 

### 3.6. Evaluation of the Cortisol Aptamer

**Figure 6:**
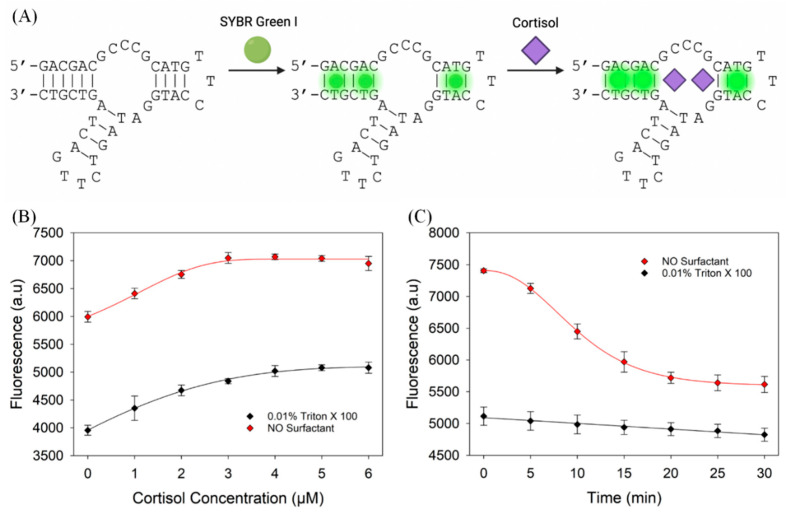
(**A**) The scheme of cortisol binding to its aptamer detected by SYBR Green I. Initially, the combination of SGI and aptamer produces weak fluorescence, and after the addition of cortisol, aptamer–cortisol binding results in enhanced fluorescence. (**B**) Titration curve of 1 µM CSS.1-42 with cortisol with and without Triton X-100. (**C**) Effect of Triton X-100 on signal stability of CSS.1-42 Apt-SGI-cortisol titration.

Finally, we used the CSS.1-42 aptamer to detect cortisol (Figure 6A) [33,34]. Our group recently showed that SGI can stain this aptamer and cortisol binding results in a moderate fluorescence increase [34]. Triton X-100 had no significant effect on the fold change in fluorescence of the system (18.0% without versus 28.4% with Triton X-100, Figure 6B), although, again, the overall fluorescence was lower in the presence of the surfactant. We then performed a 30 min fluorescence stability assay on samples containing 5 µM cortisol in a 96-well plate (Figure 6C). In the absence of Triton X-100, the fluorescence of the sample dropped rapidly in the first 10 min and decreased by 24% after 30 min. In contrast, the fluorescence signal of the system was more stable after the addition of Triton X-100 and decreased by only 5.7% in 30 min. This stable signal can make cortisol detection more accurate. 

## 4. Conclusions

Recently, many new aptamers have been reported, [33,35,36,37] and the development of reliable binding assays is ever more important. [1,2,38] In this work, we examined the stability of the fluorescence signal of various DNA staining dyes with various aptamers in both polystyrene microplates and a quartz cuvette. A time-dependent fluorescence drop was observed in most cases, especially in the microplates, attributable to the adsorption of cationic DNA-staining dyes to the walls of the vessels. We can classify the purpose of such experiments into two types. First, for qualitative measurement to answer questions such as whether a DNA sequence is an aptamer or not; using carefully designed control sequences and control target molecules is very important to confirm that the change in fluorescence is due to aptamer binding instead of other events, such as dye adsorption. Even in the presence of nonspecific dye adsorption, aptamer binding might still generate a greater signal change. Using a single aptamer sequence cannot generate reliable answers. The best control sequences would be those with a point mutation. As can be seen from this study, the fluorescence response of different DNA can be very drastic to different DNA staining dyes. So, using a totally unrelated scramble sequence is less reliable than using mutants of aptamers.

Second, for quantitative measurements to obtain values such as *K*_d_ and kinetic information, the dye adsorption problem can be mitigated using either a long waiting time with agitation or by adding a surfactant such as Triton X-100. Using this surfactant, we tested three aptamers for Hg^2+^, adenosine, and cortisol. In each case, a more stable signal was observed with the surfactant, although a target concentration-dependent fluorescence change was achieved even without the surfactant. In addition, using a quartz cuvette is recommended over a plastic microplate to achieve more accurate *K*_d_ measurement.

Overall, we articulated the signal stability problem of DNA staining dye-based aptamer binding assays and provided a few solutions to solve this problem to generate more reliable measurements and biosensors.

## Figures and Tables

**Table 1:** DNA sequences and modifications used in this work.

Name	Sequences (5′-3′)
DNA1	ACGACACGGAGGCTTAGTTTGCTAAATGGTCATGTCGT
CSS.1-42	GACGACGCCCGCATGTTCCATGGATAGTCTTGACTAGTCGTC
Ade Apt	ACCTGGGGGAGTATTGCGGAGGAAGGT
Ade Apt M2	ACCTGGGGTAGTATTGCGGAGTAAGGT
T_30_	TTTTTTTTTTTTTTTTTTTTTTTTTTTTTT

**Table 2:** Surfactant molecular weights, relevant molar concentrations at 0.01%, and critical micelle concentrations (CMC) [30,31].

Surfactant	Molecular Weight	Concentration at 0.01% (mM)	CMC (mM)
Triton X-100	646.9	0.155	0.19–0.22
TWEEN 20	522.7	0.191	0.046
SDS	288.4	0.347	7.3–8.1
CTAB	364.5	0.274	0.98

## Data Availability

Not applicable.
